# Milk Fat Globule Membrane Attenuates High-Fat Diet-Induced Obesity by Inhibiting Adipogenesis and Increasing Uncoupling Protein 1 Expression in White Adipose Tissue of Mice

**DOI:** 10.3390/nu10030331

**Published:** 2018-03-09

**Authors:** Tiange Li, Jing Gao, Min Du, Jiajia Song, Xueying Mao

**Affiliations:** 1 Beijing Advanced Innovation Center for Food Nutrition and Human Health, College of Food Science & Nutritional Engineering, China Agricultural University, Beijing 100083, China; 13126750913@163.com (T.L.); gaokeke1992@126.com (J.G.); songjiajia1208@126.com (J.S.); 2 College of Food Science and Nutritional Engineering, Key Laboratory of Functional Dairy, Ministry of Education, China Agricultural University, Beijing 100083, China; 3 Department of Animal Sciences, Washington State University, Pullman, WA 99164, USA; min.du@wsu.edu

**Keywords:** milk fat globule membrane, adipogenesis, adipose tissue, obesity, uncoupling protein 1

## Abstract

Milk fat globule membrane (MFGM), a protein-lipid complex surrounding the fat globules in milk, has many health benefits. The aim of the current study was to investigate whether MFGM could prevent obesity through inhibiting adipogenesis and promoting brown remodeling of white adipose tissue (WAT) in mice fed with high-fat diet. C57BL/6 mice were fed a normal diet (ND), high-fat diet (HFD), HFD plus MFGM at 100 mg/kg BW, 200 mg/kg BW or 400 mg/kg BW for 8 weeks. Results showed that MFGM suppressed body weight gain induced by HFD, reduced white adipose tissue (WAT) mass accompanied with the decrease in adipocyte sizes. MFGM was found to have partially improved serum lipid profiles, as well as to have suppressed HFD-induced adipogenesis as shown by reduced expression of peroxisome proliferators-activator receptor-γ (PPARγ), CCAAT/enhancer-binding protein-α (C/EBPα) and sterol regulatory element-binding protein-1c (SREBP-1c). MFGM also markedly increased the phosphorylation of AMP-activated protein kinase (AMPK) and acetyl-CoA carboxylase (ACC), showing activation of AMPK pathway. Moreover, MFGM promoted browning of inguinal WAT by upregulation the protein expression of uncoupling protein 1 (UCP1) in HFD mice. Taken together, these findings provide evidence that MFGM may protect against diet-induced adiposity by suppressing adipogenesis and promoting brown-like transformation in WAT.

## 1. Introduction

Obesity has become a public health dilemma that has steadily increased in the past few decades. It is a risk factor for various metabolic disturbances, including type 2 diabetes, dyslipidemia and cardiovascular disease [1]. It is associated with an expansion of adipose tissue due to excessive energy intake and lack of exercise, resulting in an abnormal accumulation of body fat [2].

Mammals were previously believed to have two major types of adipose tissue, called white adipose tissue (WAT) and brown adipose tissue (BAT), which showed difference in function and morphology [3]. WAT stores excessive energy as triglycerides, whereas BAT dissipates chemical energy as heat [4]. Uncoupling protein 1 (UCP1), which is specifically expressed in BAT, plays an important role in energy expenditure, and promoting its expression could reduce obesity and metabolic dysfunction [5]. Recently, “brown-like” adipocytes were identified in WAT depots in response to certain stimuli, and these adipocytes were referred to as beige or ‘brite’ (brown in white) adipocytes [6]. Similar to classic brown adipocytes, beige adipocytes in WAT are composed of multilocular/small lipid droplets and high amounts of mitochondria that contain UCP1 and can ‘burn’ fat [7]. Browning of WAT is inducible and promoted by dietary supplements such as olive oil, resveratrol and capsaicin [8]. Thus, the induction of browning in WAT represents an attractive strategy to combat obesity and its complications.

Adipogenesis is a differentiation process during which the mesenchymal precursor cells develop into mature adipocytes [9]. The differentiation of preadipocytes into adipocytes requires the sequential expression of adipogenic transcription factors, including peroxisome proliferators-activator receptor-γ (PPARγ), CCAAT/enhancer-binding protein-α (C/EBPα) and sterol regulatory element-binding protein-1c (SREBP-1c) [10]. AMP-activated kinase (AMPK) is a master regulator in control of energy homeostasis and lipid metabolism, and its activation inhibits the expression of PPARγ and C/EBPα and adipogenesis [11]. Phosphorylation of AMPK suppresses lipid synthesis by inactivating lipogenic genes in adipose tissue, such as acetyl-CoA carboxylase (ACC), a key enzyme of lipogenesis that synthesizes malonyl-coenzyme A (malonyl-CoA) from acetyl-CoA [12]. Therefore, inhibition of preadipocyte proliferation and differentiation might be an effective strategy for preventing obesity and its associated metabolic disorders.

Milk fat globule membrane (MFGM), a tri-layer membrane consisting of proteins and lipids, is derived from the apical surface of mammary epithelial cells, which surrounds the fat globules in milk [13]. Several benefits of MFGM components, such as anti-cancer, antibacterial and anti-inflammatory, have been reported [14]. Recently, MFGM has been attracting much attention in protection against obesity and related metabolic disorders. Milk sphingomyelin, one of the major polar lipids in MFGM, significantly inhibited intestinal lipid absorption and reduced serum and liver lipid levels in obese/diabetic KK-Ay mice [15]. Milk sphingomyelin also improved lipid metabolism by reducing hepatic triglycerides and inducing gene expression associated with cholesterol biosynthesis in high-fat diet-fed mice [16]. In addition to functions attributed to its individual components, clinical trials in overweight and obese individuals also showed that the addition of a dairy fraction rich in MFGM reduced postprandial cholesterol levels, improved insulin response and increased the production of anti-inflammatory markers, which attenuated the negative effects of a high-saturated-fat meal [17]. In addition, milk fat globule membrane in the diet of young mice reduced body fat accumulation against obesity in later life [18]. However, whether MFGM could alleviate obesity by altering adipogenesis or brown remodeling of WAT is not fully understood.

The objective of the present study was to investigate the effect of MFGM on adipogenesis and WAT browning in high-fat diet-induced mice.

## 2. Materials and Methods

### 2.1. Materials

Lacprodan^®^ MFGM-10 was obtained from Arla Co. (Sønderhøj, Viby J, Denmark). Antibodies against C/EBPα, SREBP-1c and β-actin were purchased from Bioss Inc. (Bios, Beijing, China) and antibodies against PPARγ, AMPK, p-AMPK, ACC and p-ACC were purchased from Cell Signaling Technology (Beverly, MA, USA). The antibody against UCP1 was obtained from Abcam (Cambridge, UK).

### 2.2. Animals and Treatments

Male C57BL/6J mice (5-week-old) were purchased from Beijing Vital River Laboratory Animal Technology Co., Ltd. (Beijing, China). All mice were housed under 12 h light/dark cycle and a constant temperature (24 °C). After one-week acclimation, mice were subsequently divided into two groups randomly. The mice in the different groups were fed, respectively, a normal chow diet (ND group, KeAoXieLi Feed Co., Ltd., Beijing, China) (*n* = 10) or a high-fat diet (KeAoXieLi Feed Co., Ltd., Beijing, China) (*n* = 40) for 8 weeks. The normal chow diet provided 3.01 kcal/g of energy (25.75% calories from protein, 13.40% calories from fat and 60.85% calories from carbohydrate), whereas the high-fat diet provided 5.24 kcal/g of energy (20.05% calories from protein, 60.08% calories from fat and 20.04% calories from carbohydrate). Then mice fed high-fat diet were randomly divided into 4 groups (*n* = 10/group) and fed with high-fat diet (HFD group, *n* = 10), high-fat diet with MFGM at 100 mg/kg BW (M100 group, *n* = 10), 200 mg/kg BW (M200 group, *n* = 10) and 400 mg/kg BW (M400 group, *n* = 10) for another 8-week. Body weight and food intake were monitored every other day. At the end of the experimental period, mice were anesthetized after fasting and blood was taken to collect serum. The adipose tissues were removed, rinsed with physiological saline solution, weighed and stored at −80 °C until analysis. The animal protocols (Approval No. KY160018) were approved by the Animal Experimentation Committee of China Agricultural University (Beijing, China).

### 2.3. Western Blot Analysis

Adipose tissues were homogenized in an ice cold lysis buffer (Beyotime Biotech, Haimen, Jiangsu, China) containing 1% NP-40, 0.5% deoxycholate, 0.1% SDS and 1% protease inhibitor cocktail (Roche Diagnostics, Mannheim, Germany). The homogenate was centrifuged at 12,000× *g* for 10 min at 4 °C and the supernatant was collected. Protein concentration in samples was determined using BCA Protein Assay Reagent (Beyotime Biotech, Haimen, Jiangsu, China). The 20 µg of protein of each sample was electrophoretically separated in 7.5% to 12.5% sodium dodecyl sulfate polyacrylamide gel and then transferred to polyvinylidene diflouride membrane (Millipore, Billerica, MA, USA). Membranes were blocked with 5% (*w*/*v*) skimmed milk powder in tris-buffered saline supplemented with 0.05% (*v*/*v*) Tween 20 (TBST) for 2 h and then incubated with primary antibodies overnight at 4 °C. The membranes were next incubated with horseradish-peroxidase-conjugated secondary antibodies at room temperature for 1 h. The bands were visualized with enhanced chemiluminescence (ECL) reagents (Millipore, Billerica, MA, USA), and quantified densitometrically using the software ImageJ 1.47v (Wayne Rasband, Bethesda, MD, USA).

### 2.4. Total RNA Isolation and Real-Time Reverse Transcriptase Polymerase Chain Reaction (RT-PCR) Analysis

Total RNA from epididymal WAT was extracted with Trizol reagent (Invitrogen, Waltham, MA, USA) according to the manufacturer’s instructions. The cDNA was synthesized from total RNA (3 µg) in a 25 μL reaction volume using the TIANScript RT Kit (Tiangen Biotech, Beijing, China). Real-time PCR was performed using 1 μL cDNA with the SYBR Premix Ex Taq RT-PCR kit (Takara, Otsu, Shiga, Japan) on Techne Quantica real-time PCR detection system (Techne, Staffordshire, UK). The thermal profile settings were starting with a denaturation at 95 °C for 180 s followed by 40 cycles of 95 °C for 30 s, 60 °C for 30 s, 72 °C for 30 s. The SYBR green fluorescence was read at the end of each extension step (72 °C). Relative expression levels of the mRNA of the target genes were normalized to 18S mRNA levels. PCR sequences of various genes are presented in Table 1.

### 2.5. Measurement of Serum Biochemical Parameters

Serum was separated after blood sampling by centrifugation at 1500× *g* for 15 min. The concentrations of serum triglyceride (TG), total cholesterol (TC), low-density lipoprotein (LDL-C), high-density lipoprotein (HDL-C) cholesterol and free fatty acids (FFA) were determined using commercially diagnostic kits supplied by R&D Systems (Minneapolis, MN, USA) according to the manufacturer’s instructions.

### 2.6. Histological Analysis

Epididymal WAT was dissected, washed in saline and immediately fixed in 4% paraformaldehyde. Fixed tissues were embedded in paraffin and 4 μm sections were prepared, stained with hematoxylin and eosin (H&E) for general morphological observations. Images were taken at 200× magnification. The adipocyte sizes of white adipose tissue were quantitated using Photoshop CS6 (Adobe Systems Inc., San Jose, CA, USA). Adipocyte volume (v) was calculated as the formulation v = πd^3^/6 and the adipocyte weight equal volume multiplied by density (about 0.915 g/cm^3^) [19]. Adipocyte number was determined by dividing the epididymal WAT weight by the average adipocyte weight.

### 2.7. Statistical Analysis

All data were presented as means ± S.E.M. (the standard error of the mean). Significance for multiple comparisons was determined using one-way ANOVA followed by Duncan corrections. Statistical significance was established at *p* < 0.05 level. All statistical analyses were performed using SPSS 17.0 (SPSS Inc., Chicago, IL, USA).

## 3. Results

### 3.1. MFGM Suppresses HFD-Induced Increases in Body Weight of Mice

**Figure 1:**
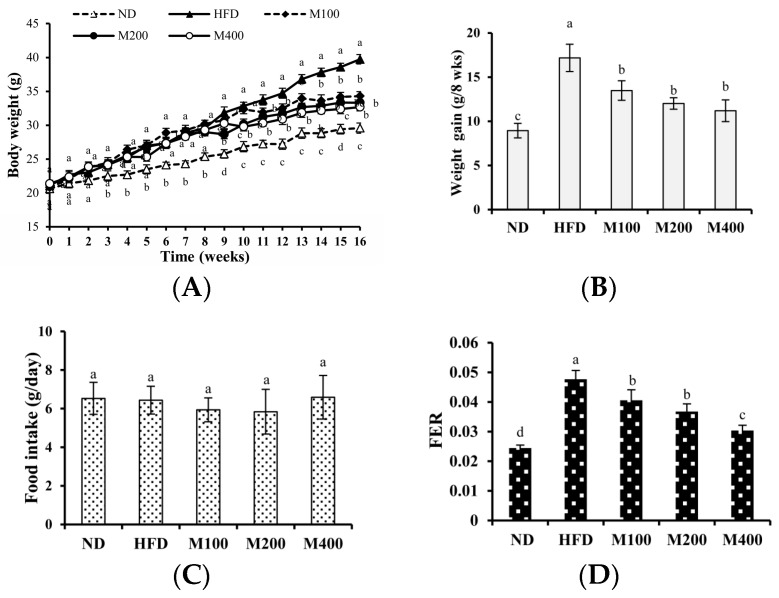
Effect of MFGM on the body weight gain in HFD-fed mice. Mice were fed with a normal chow diet (ND), a high-fat diet (HFD), high-fat diet supplemented with MFGM at 100 mg/kg BW (M100), 200 mg/kg BW (M200) or 400 mg/kg BW (M400) for eight weeks. Changes in the (**A**) body weight; (**B**) body weight gain; (**C**) food intake and (**D**) food efficiency ratio (FER). FER = Body weight gain (g)/food intake during trial period (g). Means ± SE (*n* = 6). Mean values with different letters indicate statistical significance (*p* < 0.05).

As shown in Figure 1A, at the beginning of the study (0 week), the initial body weights of mice assigned for ND (20.63 ± 0.56 g), HFD (21.05 ± 0. 76 g), M100 (20.81 ± 0.65 g), M200 (21.30 ± 0.57 g) and M400 (21.37 ± 0.47 g) groups were the same. At week 8, the final average body weights of the HFD, M100, M200 and M400 groups were significantly higher than that of the ND group, indicating that high-fat diet had induced obesity. From week 9 to the end of the study, body weight gain in M100, M200 and M400 group was reduced by 21.52%, 30.07% and 34.82%, respectively, compared to that in HFD group (Figure 1B). The food intake in mice of all groups was also assessed. As shown in Figure 1C,D, compared with the HFD group, MFGM (100, 200 or 400 mg/kg) treatment significantly decreased the food efficiency ratio (FER) with no alteration in food intake, suggesting the reduced weight gain is not a result of reduced calorie intake.

### 3.2. MFGM Prevents Alterations in Adipose Tissue Mediated by HFD of Mice

**Figure 2:**
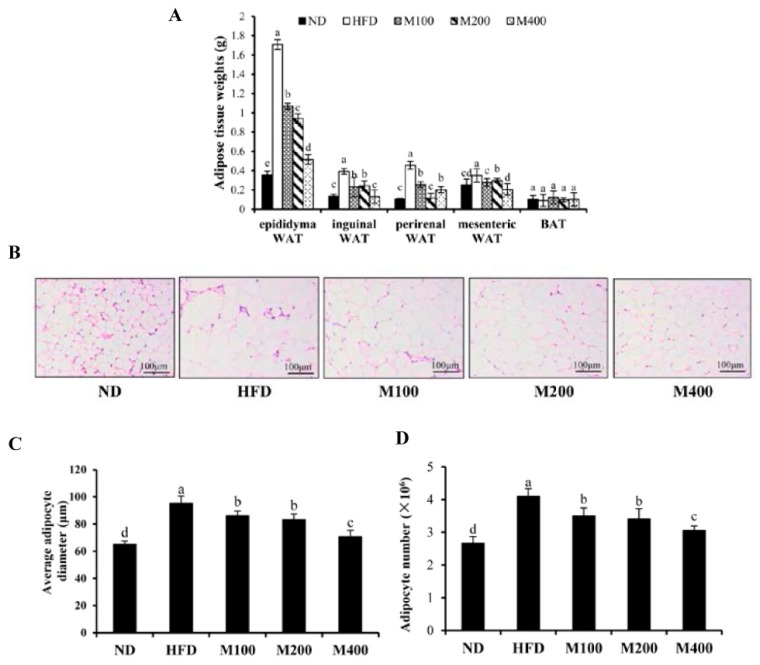
Effect of MFGM on adipose mass gain. (**A**) Changes in fat pad weights of epididymal white adipose tissue (WAT), inguinal WAT, perirenal WAT, mesenteric WAT and brown adipose tissue (BAT); (**B**) Representative hematoxylin and eosin staining of epididymal WAT; (**C**) Average adipocyte diameter of epididymal WAT. Scale bar: 100 µm, 200×; (**D**) The calculated adipocyte number in epididymal WAT. Means ± SE (*n* = 6). Mean values with different letters indicate statistical significance (*p* < 0.05).

To investigate whether the decreased body weight gain was due to the reduction in adipose tissue mass, the fat pad weights were measured. As shown in Figure 2A, the feeding of HFD significantly increased fat pad weights of epididymal WAT, inguinal WAT, perirenal WAT and mesenteric WAT compared with that of the ND group. MFGM supplementation significantly decreased the HFD-induced gains of WAT weights, while BAT weight did not change significantly. Histological sections from the epididymal WAT of HFD group showed a greater adipocyte diameter as compared with ND group, whereas adipocyte size in M100, M200 and M400 group were clearly smaller than that of HFD group (Figure 2B,C). Adipose mass was correlated with body weight in the mice fed MFGM, suggesting that the MFGM-induced decrease in body weight could be attributed to a reduction in adipose tissue weights. Besides, based on the average adipocyte diameter in epididymal WAT and the weight of epididymal WAT, the total number of adipocyte was calculated. Results showed that MFGM significantly decreased adipocyte number in epididymal WAT compared with HFD group (Figure 2D), suggesting that MFGM may inhibit adipogenesis and hyperplasia of epididymal WAT.

### 3.3. MFGM Prevents Alterations in Adipose Tissue Mediated by HFD of Mice

As shown in Table 2, compared to the ND group, high-fat diet significantly increased serum levels of TG, TC, HDL-C, LDL-C, FFA and reduced the ratio of HDL-C/LDL-C. MFGM supplementation significantly lowered not only the TG and TC but also the FFA levels compared to those in the HFD group. The levels of serum HDL-C and LDL-C were markedly decreased, and the ratio of HDL-C/LDL-C was upregulated by the MFGM treatment compared to those in the HFD group.

### 3.4. MFGM Regulates the Expression of Adipogenesis-Related Genes and Proteins in Epididymal WAT of HFD-Fed Mice

**Figure 3:**
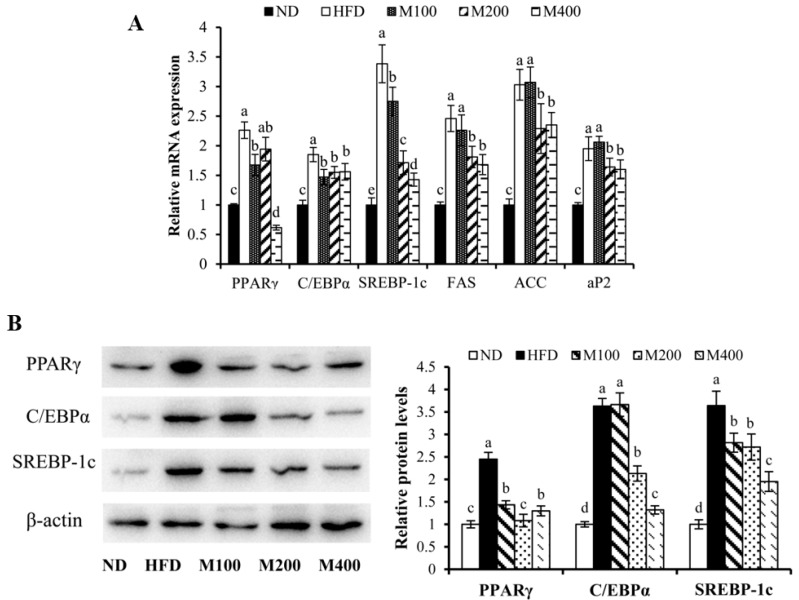
Effect of MFGM on the expression of adipogenesis-related genes and proteins in epididymal WAT. (**A**) Relative mRNA expression levels of adipogenic genes were measured using RT-PCR; (**B**) Adipogenesis related protein expressions were measured by western blot analysis. Protein expressions were quantified densitometrically using the software ImageJ 1.47v (Wayne Rasband, Bethesda, MD, USA), and were arbitrary units after correction for loading differences by measuring the amount of β-actin. Means ± SE (*n* = 3). Mean values with different letters indicate statistical significance (*p* < 0.05).

To investigate whether MFGM regulates adipogenesis in epididymal WAT, the expression of adipogenesis-related genes and proteins were measured by RT-PCR and western blot analysis, respectively. Mice fed HFD showed markedly increased mRNA expression of PPARγ, C/EBPα, SREBP-1c, fatty acid synthase (FAS), ACC and adipocyte fatty acid-binding protein (aP2) (Figure 3A), while MFGM supplementation significantly reduced the mRNA levels of these genes compared to the HFD group. As shown in Figure 3B, the protein expression of PPARγ, C/EBPα and SREBP-1c in epididymal WAT of mice fed HFD was elevated by 1.45-fold, 2.63-fold and 2.64-fold, respectively, compared with those of normal mice. However, PPARγ, C/EBPα and SREBP-1c protein expressions were strongly decreased in mice fed HFD plus MFGM compared to those in HFD-fed mice. MFGM supplementation (400 mg/kg) decreased PPARγ, C/EBPα and SREBP-1c protein expressions by approximately 46.94%, 63.63%, 46.43%, respectively, compared with those of mice fed HFD. These results suggest that the MFGM-mediated decrease in adipose tissue weight could be attributed to a reduction in adipogenic differentiation and hypertrophy, which correlated with improved circulatory lipid profiles.

### 3.5. MFGM Activates AMPK Pathway in Epididymal WAT of HFD-Fed Mice

**Figure 4:**
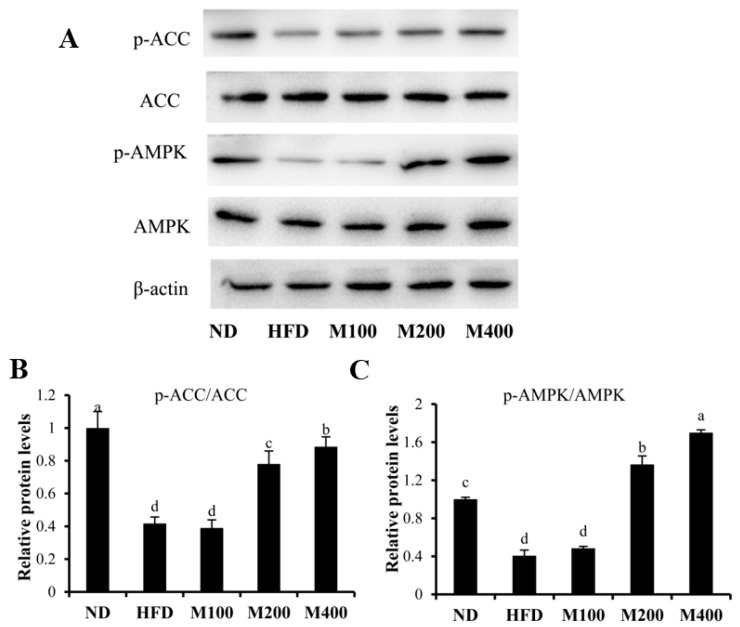
Effect of MFGM on the activation of AMPK pathway in epididymal WAT. The protein expressions of AMPK, p-AMPK, ACC, p-ACC (**A**) and relative protein levels of p-ACC/ACC (**B**) and p-AMPK/AMPK (**C**) were measured by western blot analysis. Protein expressions were quantified densitometrically using the software ImageJ 1.47v (Wayne Rasband, Bethesda, MD, USA), and were arbitrary units after correction for loading differences by measuring the amount of β-actin. Means ± SE (*n* = 3). Mean values with different letters indicate statistical significance (*p* < 0.05).

To investigate whether MFGM regulates lipogenesis by activating AMPK pathway, the contents of p-AMPK and p-ACC were assessed in epididymal WAT. High-fat diet suppressed the phosphorylation of AMPK, while MFGM treatment restored AMPK phosphorylation (Figure 4A). The p-AMPK/AMPK ratio in M400 group increased by approximately 3.19-fold compared to that in HFD group (Figure 4B). Phosphorylation of ACC, a key enzyme of lipogenesis which synthesizes malonyl-CoA from acetyl-CoA, was inhibited by HFD, while MFGM treatment increased ACC phosphorylation. The ratio of p-ACC/ACC in M400 group increased by about 1.12-fold compared with that in mice fed HFD (Figure 4C).

### 3.6. MFGM Upregulates UCP1 Protein Expression in Inguinal WAT and BAT of HFD-Fed Mice

**Figure 5:**
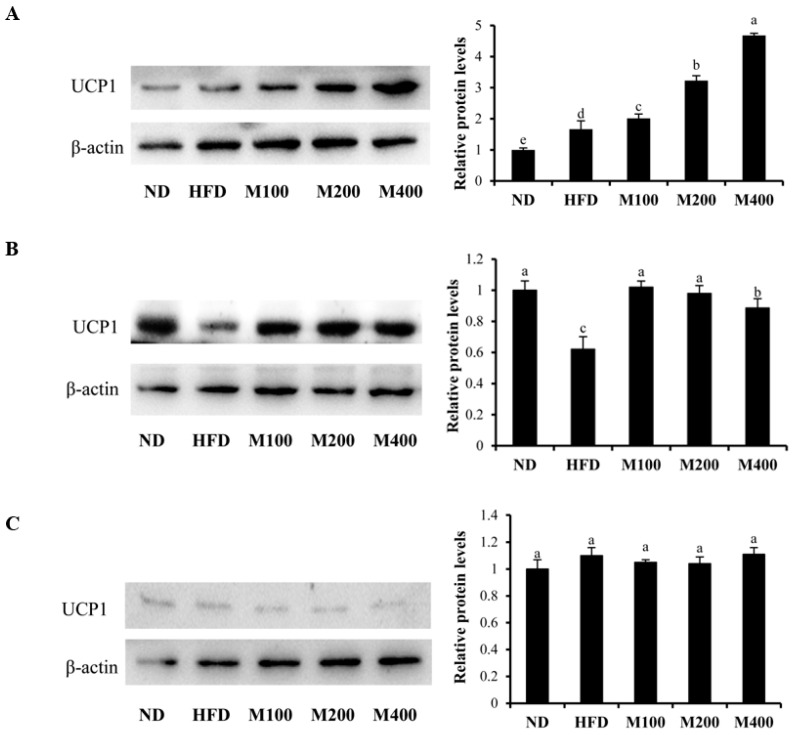
Effect of MFGM on the upregulation of UCP1 protein expression in inguinal WAT, epididymal WAT and BAT. a–c: UCP1 protein expression in BAT (**A**); inguinal WAT (**B**); and epididymal WAT (**C**) was determined by western blot analysis. Protein expressions were quantified densitometrically using the software ImageJ 1.47v (Wayne Rasband, Bethesda, MD, USA), and were arbitrary units after correction for loading differences by measuring the amount of β-actin. Means ± SE (*n* = 3). Mean values with different letters indicate statistical significance (*p* < 0.05).

To determine whether the inhibitory effect of MFGM on HFD-induced obesity is also due to the browning of WAT, the protein expression of UCP1 in epididymal WAT, inguinal WAT and BAT were also determined. As shown in Figure 5A, MFGM treatment significantly upregulated the expression of UCP1 in BAT compared to that of the mice only fed high fat-diet. In addition, MFGM also increased the UCP1 content in inguinal WAT (Figure 5B), while MFGM had no effect on UCP1 expression in epididymal WAT (Figure 5C). This suggests that MFGM not only enhanced BAT activity but also promoted browning of inguinal WAT.

## 4. Discussion

Obesity and metabolic diseases are rapidly becoming major global health problems. Consequently, research to identify natural products for the prevention and treatment of obesity is gaining more and more attention [20]. MFGM is a bioactive component of milk which possesses various health benefits but its anti-obesity effect remains unclear. In the current study, we found that MFGM suppressed HFD-induced obesity in mice, which is attributable to the inhibition of adipogenic differentiation by the activation of AMPK pathway. MFGM also upregulated the UCP1 protein expression in both BAT and inguinal WAT. These findings suggest that MFGM supplementation could mitigate obesity by inhibiting adipogenesis, improving browning of WAT and increasing BAT activity.

Obesity, defined as an excess abnormal accumulation of body fat mass, leads to a series of metabolic complications [21]. C57BL/6 mice fed with high-fat diet are widely used to assess the development of obesity [22]. Chronic administration of a high-fat diet to C57BL/6 mice resulted in obesity development [23]. In the present study, mice fed with HFD induced obesity while MFGM supplement mitigated the body weight gain without influencing the food intake.

Adipose tissue is an important contributor to energy storage and energy utilization [24]. However, excess adipose tissue can have many negative effects on an individual’s health [25]. Over-nutrition leads to the expansion of adipose tissue which is associated to the increase in the size and number of adipocytes, and eventually causes adipocyte hypoxia, inflammation and insulin resistance [26]. In our study in mice, high-fat diet increased fat pad weights and the size of adipocytes with the elevation of serum TG levels. After administration of MFGM, the mice had smaller adipocytes in epididymal WAT and the TG levels in serum was closer to that in the ND group. These observations are consistent with previous reports that high-fat diet-induced obese mice exhibited a modest hypertriglyceridaemia characterized by increases in TC, LDL-C and HDL-C levels and reduction in the ratio of HDL-C/LDL-C. MFGM treatment also lowered the levels of TC, HDL-C and LDL-C in serum and up-regulated the ratio of HDL-C/LDL-C. These observations suggested that MFGM reduces weight gain is partially due to the alleviation of lipid accumulation in WAT and the improvement of lipid profiles.

Adipogenesis can be separated into adipogenic commitment and differentiation [27]. Several transcription factors, including PPARγ, C/EBPa and SREBP-1c, play roles during the differentiation of pre-adipocytes [28]. Among these factors, PPARγ has been recognized as a key nuclear receptor transcription factor in the control of adipogenesis. It regulates the expressions of the genes related to fatty acid synthesis, oxidation and adipogenesis, such as ACC, FAS and aP2 [29]. PPARγ mediates high-fat diet-induced adipocyte differentiation, whereas PPARγ-deficient mice were protected from adipocyte hypertrophy under a high-fat diet [30]. C/EBPα is another transcription factor and promotes adipogenesis in the early phase of preadipocyte differentiation. PPARγ and C/EBPα reinforce each other to drive adipogenic differentiation [31]. Besides, SREBP-1c can activate PPARγ by increasing its expression and by stimulating the production of its ligands [32]. We found that the mRNA expression levels of PPARγ, C/EBPα, SREBP-1c, FAS, aP2 and ACC were remarkably increased in mice fed high-fat diet, but these genes were significantly decreased when treated with different concentrations of MFGM, showing its inhibitory effects on adipogenic differentiation.

AMP-activated protein kinase (AMPK) is a key signaling protein in the regulation of cellular energy homeostasis, lipid metabolism and mitochondrial biogenesis [33]. AMPK activation in adipose tissue suppresses PPARγ and inhibits adipogenesis, which consequently reduces fat accumulation [34]. The phosphorylation of AMPK also enhances fatty acid oxidation by the inactivation of lipogenic enzymes such as ACC [35]. Dietary bioactive compounds such as sulforaphane [11], oxyresveratrol [36] and green tomato extract [37] prevent high-fat diet-induced obesity and reduce lipid accumulation by stimulating AMPK activity. Consistent with these reports, MFGM administration induced a statistically significant increase in the phosphorylation of AMPK and ACC accompanied by the upregulation of PPARγ, C/EBPα and SREBP-1c protein expression.

Browning of WAT is an attractive strategy for combating obesity and associated metabolic diseases [7]. Beige adipocytes express UCP1 and other protein machinery involved in thermogenesis. UCP1, which dissipates the proton gradient across the mitochondrial inner membrane to produce heat, is generally considered as a crucial marker of browning of WAT and BAT activity. The browning of WAT is inducible and promoted by many factors including adipokines, myokines, cytokines, and dietary factors [8]. Curcumin, a naturally occurring curcuminoid of turmeric with anti-obesity effect, induces a brown-like phenotype in both 3T3-L1 and primary white adipocytes [38]. Berberine increased the expression of UCP1 and other thermogenic genes in white and brown adipose tissue of mice [6]. Consistent with these findings, MFGM also significantly upregulated the protein expression of UCP1 in BAT and inguinal WAT, suggesting the potential role of MFGM on BAT activity and browning of inguinal WAT. However, the exact mechanism of MFGM on the browning of WAT needs further investigation.

## 5. Conclusions

In summary, our results suggested that MFGM supplement significantly reduced obesity in HFD mice. The adipose tissue mass, the size of adipocytes and serum lipid levels were decreased by MFGM consumption. MFGM suppressed the protein and mRNA expression of PPARγ, C/EBPα and SREBP-1c and activating AMPK pathway, which correlate with suppression of adipogenic differentiation. MFGM also induced the formation of brown-like adipocytes as indicated by upregulating the protein expression of UCP1. These observations show that dietary MFGM has the potential to prevent or treat obesity.

## Figures and Tables

**Table 1:** Primers used for Real-Time Quantitative Polymerase Chain Reaction (RT-PCR).

Gene	Gene Bank No.	Forward Sequence (5′–3′)	Reverse Sequence (5′–3′)
PPARγ	NM_011146.3	AGCTCCAAGAATACCAAAGTGCGAT	AGGTTCTTCATGAGGCCTGTTGTAGA
C/EBPα	NM_007678.3	AAACAACGCAACGTGGAGA	GCGGTCATTGTCACTGGTC
SREBP-1c	NM_011480.4	CCCTGTGTGTACTGGCCTTT	TTGCGATGTCTCCAGAAGTG
FAS	NM_007988.3	AGAGATCCCGAGACGCTTCT	GCCTGGTAGGCATTCTGTAGT
ACC	NM_133360.2	GAATCTCCTGGTGACAATGCTTATT	GGTCTTGCTGAGTTGGGTTAGCT
aP2	NM_024406.2	CATGGCCAAGCCCAACAT	CGCCCAGTTTGAAGGAAATC

**Table 2:** Effect of MFGM on serum lipid profile in HFD-fed mice.

Parameters	Dietary Group				
	ND	HFD	M100	M200	M400
TG (mmol/L)	0.17 ± 0.04 ^c^	0.42 ± 0.03 ^a^	0.37 ± 0.02 ^a^	0.27 ± 0.03 ^b^	0.25 ± 0.04 ^b^
TC (mmol/L)	2.58 ± 0.42 ^c^	5.58 ± 0.61 ^a^	4.28 ± 0.24 ^b^	3.97 ± 0.33 ^b^	3.71 ± 0.41 ^b^
HDL-C (mmol/L)	1.35 ± 0.20 ^c^	2.25 ± 0.34 ^a^	1.93 ± 0.11 ^a,b^	1.69 ± 0.07 ^b,c^	1.61 ± 0.13 ^b,c^
LDL-C (mmol/L)	0.14 ± 0.04 ^e^	0.41 ± 0.02 ^a^	0.33 ± 0.02 ^b^	0.26 ± 0.03 ^c^	0.21 ± 0.01 ^d^
FFA (mmol/L)	1.37 ± 0.12 ^d^	2.74 ± 0.33 ^a^	2.23 ± 0.19 ^b^	1.79 ± 0.15 ^c^	1.74 ± 0.21 ^c^

Means ± SE (*n* = 6). Mean values with different letters indicate statistical significance (*p* < 0.05).
